# Look to Your Pumps

**Published:** 1871-02

**Authors:** 


LOOK TO YOUR PUMPS AND WATER-PIPES.—Galvanized iron is supposed by many to be perfectly safe for use in pipes for conveying water, as it is often used in the endless-chain pump so common in some farming districts. Dr. Jackson, the Boston chemist, says, in regard to galvanized iron, that "it is protected by the more ready oxidation of the zinc, and the oxide of zinc is largely dissolved by water, rendering it unwholesome. In several instances I have detected large proportiens of oxide of zinc in water that has remained over night in galvanized iron pipes; and in one instance, a gentleman who brought me such water said it produced in him much nausea. The water analyzed was found to be highly charged with oxide of zinc. It is well known that when zinc-covered roofs were first introduced in Boston, and rain-water from them was used for washing, that the washer-women complained that the water made the skin of their hands crack, and the rain-water from zinc-roofs was hard, and decomposed the soap. It is also known that the French Government has recently forbidden the use of galvanized iron water-tanks in their ships, on account of the injurious effects of the dissolved zinc on the health of the men." Our readers will do well to remember this, as unprincipled dealers will deny these statements in order to sell their wares. Galvanized iron is sold at a great profit.—*Herald of Health.*

